# 577. Acceptability, Feasibility and Appropriateness of the Mobile Outreach Retention and Engagement (MORE) Program and Home Long-Acting Cabotegravir/Rilpivirine (CAB+RPV LA) Injections for People with HIV (PWH) Experiencing Adherence Challenges at Whitman-Walker Health in Washington D.C

**DOI:** 10.1093/ofid/ofaf695.186

**Published:** 2026-01-11

**Authors:** Megan Dieterich, Rupa Patel, Maria Rybicki-Newman, Keyerra Richardson, Eleanor Sarkodie, Janelle Schrag, Robert Bangert, Chris Kubaska, Lauren Brittingham, Meghan Davies, Namrata Shah, H Jonathon Rendina

**Affiliations:** Whitman-Walker Institute, Washington DC, District of Columbia; Whitman-Walker Institute, Washington DC, District of Columbia; Whitman-Walker Institute, Washington DC, District of Columbia; The Institute for Health Research & Policy at Whitman-Walker, Washington, District of Columbia; Whitman-Walker Institute, Washington DC, District of Columbia; Whitman-Walker Health, Washington, District of Columbia; Whitman-Walker Health, Washington, District of Columbia; Whitman-Walker Health, Washington, District of Columbia; Whitman-Walker Health, Washington, District of Columbia; Whitman-Walker Institute, Washington DC, District of Columbia; Whitman-Walker Institute, Washington DC, District of Columbia

## Abstract

**Background:**

Long-acting injectable (LAI) Cabotegravir/Rilpivirine demonstrated trial superiority for PWH with adherence challenges; strategies to optimize therapy remain underexplored. We report implementation outcomes for the Mobile Outreach Retention and Engagement Program (MORE) and home injections for PWH on CAB+RPV LA at Whitman-Walker Health.

**Figure:**
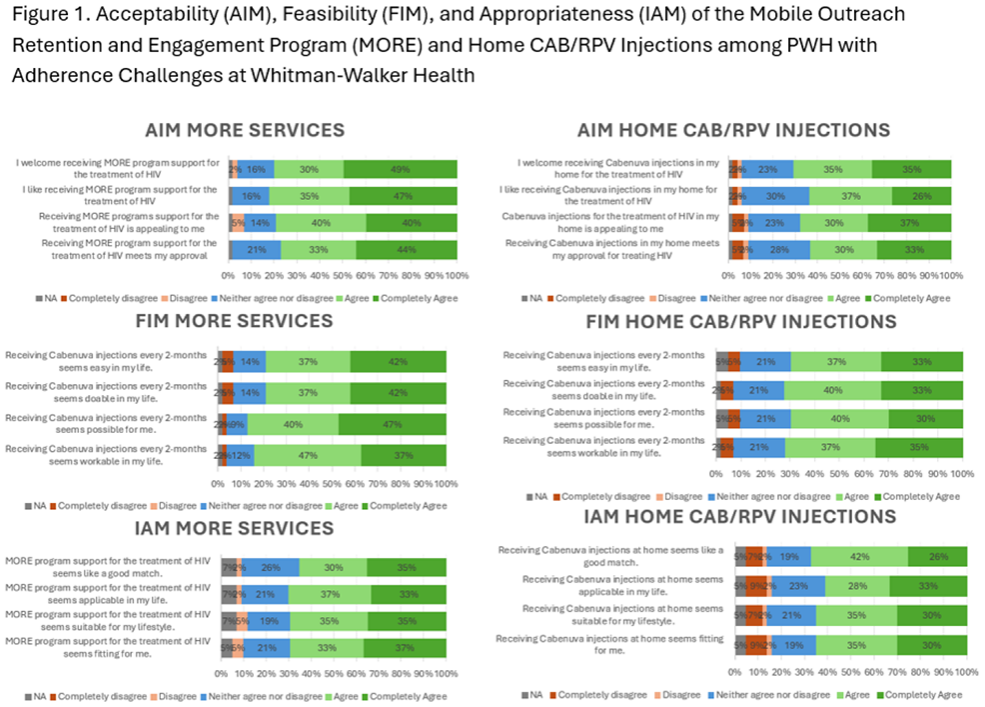

**Methods:**

MORE offers mobile care navigator support, transportation, text messaging, and optional home medical/phlebotomy/injection visits to PWH starting CAB+RPV LA with VL >200 copies/mL and/or no medical visit in the past six months. We conducted a baseline implementation assessment among 45 MORE participants, from 11/2024-2/2025, of MORE and home CAB+RPV LA injections using validated measures: Acceptability of Intervention Measure (AIM), Feasibility of Intervention Measure (FIM), and Intervention Appropriateness Measure (IAM). Each measure has 4 items rated on a 5-point Likert scale. Agreement is defined as the cumulative percentage of participants selecting “agree” or “strongly agree.” A subsample (n=25) participated in qualitative interviews.

**Results:**

Participants had a median age of 44 years, 86% were Black/AA, 38% cis women, 13% transwomen, and 80% had public insurance. MORE acceptability was ≥77%, feasibility was ≥79%, and appropriateness was ≥65%. Home injection acceptability was ≥63%, feasibility was ≥70%, and appropriateness was ≥61%. Qualitative findings for MORE were that familiarity with care teams enhanced acceptability, program flexibility improved feasibility, and tailored services promoted appropriateness. Views about home injections varied, with perceptions of how privacy could be maintained, and the safety of medical procedures in the home influencing acceptability and appropriateness. Flexibility between home and clinic injections improved feasibility. Sixty-seven percent (30/45) of participants were willing to receive home injections and 31% (14/45) received at least one home visit.

**Conclusion:**

Home CAB+RPV LA injections delivered through a support program were acceptable, feasible, and appropriate among PWH with adherence challenges. Incorporating similar flexible, patient-centered home LAI interventions into health centers may help address unmet needs of PWH nationwide and globally.

**Disclosures:**

All Authors: No reported disclosures

